# Myeloperoxidase-induced modification of HDL by isolevuglandins inhibits paraoxonase-1 activity

**DOI:** 10.1016/j.jbc.2021.101019

**Published:** 2021-07-29

**Authors:** Geetika Aggarwal, Linda S. May-Zhang, Valery Yermalitsky, Sergey Dikalov, Maxim A. Voynov, Venkataraman Amarnath, Valentina Kon, MacRae F. Linton, Kasey C. Vickers, Sean S. Davies

**Affiliations:** 1 Department of Pharmacology, Vanderbilt University, Nashville, Tennessee, USA; 2 Division of Clinical Pharmacology, Department of Medicine, Vanderbilt University Medical Center, Nashville, Tennessee, USA; 3 Department of Chemistry, North Carolina State University, Raleigh, North Carolina, USA; 4 Division of Nephrology and Hypertension, Department of Pediatrics, Vanderbilt University Medical Center, Nashville, Tennessee, USA; 5 Atherosclerosis Research Unit, Division of Cardiovascular Medicine, Department of Medicine, Vanderbilt University Medical Center, Nashville, Tennessee, USA

**Keywords:** paraoxonase 1 (PON1), isolevuglandin (IsoLG), myeloperoxidase (MPO), high-density lipoprotein (HDL), atherosclerosis, ^●^NO_2_, nitrogen dioxide, dHDL, dextran-isolated HDL, EPR, electron paramagnetic resonance, FH, familial hypercholesterolemia, HDL, high-density lipoprotein, hisPON1, 6xHis-tagged recombinant human PON1, IsoLGs, isolevuglandins, MDA, malondialdehyde, mol eq, molar equivalents, MPO, myeloperoxidase, ONE, 4-oxo-nonenal, PON1, paraoxonase 1, SCA, succinaldehyde, TBBL, 5-thiobutyl butyrolactone, tBHP, tert-butyl-hydroperoxide, TM10-H, 4-decyloxy-1-hydroxy-2,2,6,6-tetramethylpiperidine hydrochlorid, uHDL, ultracentrifugation-isolated HDL

## Abstract

Reduced activity of paraoxonase 1 (PON1), a high-density lipoprotein (HDL)-associated enzyme, has been implicated in the development of atherosclerosis. Post-translational modifications of PON1 may represent important mechanisms leading to reduced PON1 activity. Under atherosclerotic conditions, myeloperoxidase (MPO) is known to associate with HDL. MPO generates the oxidants hypochlorous acid and nitrogen dioxide, which can lead to post-translational modification of PON1, including tyrosine modifications that inhibit PON1 activity. Nitrogen dioxide also drives lipid peroxidation, leading to the formation of reactive lipid dicarbonyls such as malondialdehyde and isolevuglandins, which modify HDL and could inhibit PON1 activity. Because isolevuglandins are more reactive than malondialdehyde, we used *in vitro* models containing HDL, PON1, and MPO to test the hypothesis that IsoLG formation by MPO and its subsequent modification of HDL contributes to MPO-mediated reductions in PON1 activity. Incubation of MPO with HDL led to modification of HDL proteins, including PON1, by IsoLG. Incubation of HDL with IsoLG reduced PON1 lactonase and antiperoxidation activities. IsoLG modification of recombinant PON1 markedly inhibited its activity, while irreversible IsoLG modification of HDL before adding recombinant PON1 only slightly inhibited the ability of HDL to enhance the catalytic activity of recombinant PON1. Together, these studies support the notion that association of MPO with HDL leads to lower PON1 activity in part *via* IsoLG-mediated modification of PON1, so that IsoLG modification of PON1 could contribute to increased risk for atherosclerosis, and blocking this modification might prove beneficial to reduce atherosclerosis.

Paraoxonase 1 (PON1) is a 45-kDa calcium-dependent enzyme that associates with high-density lipoprotein (HDL) (1). PON1 is a lipolactonase (2, 3, 4, 5, 6, 7) that also exerts antiperoxidation activity by catalyzing the hydrolysis of lipid peroxides (8). Deletion of *Pon1* in the atherosclerosis-prone *Apoe*^*−/−*^ mice (*Apoe*^*−/−*^; *Pon1*^*−/−*^ mice) exacerbated atherosclerosis compared with *Apoe*^*−/−*^; *Pon1*^*+/+*^mice (9). In contrast, transgenic expression of human *PON1* (*via* adenovirus-mediated transfer) in *Ldlr*^*−/−*^; *ob/ob* mice reduced atherosclerosis (10). Reductions in PON1 enzymatic activity in patients with cardiovascular disease have been observed in numerous studies (3, 11, 12, 13, 14, 15, 16).

Two key polymorphisms in the PON1 gene (L55M and Q192R polymorphisms) alter its expression and activity and have been associated, albeit inconsistently, with risk for cardiovascular diseases (17, 18, 19, 20). Such inconsistent genetic effects might arise if post-translational modification of PON1 also plays a significant role in reducing PON1 activity. Association of myeloperoxidase (MPO) with HDL has been implicated in the loss of PON1 activity in cardiovascular diseases (21). In the presence of chloride and nitrite ions, MPO generates hypochlorous acid and nitrogen dioxide (^●^NO_2_), respectively, both of which can directly modify tyrosine residues (22, 23). Association of MPO with HDL leads to chlorotyrosine modification of PON1 tyrosine71, which has been documented in PON1 isolated from human atherosclerotic plaque (21). However, MPO may also inactivate PON1 through additional post-translational modification because ^●^NO_2_ generated by MPO in the presence of nitrite induces lipid peroxidation (23, 24, 25), leading to the formation of reactive lipid dicarbonyls such as malondialdehyde (MDA), succinaldehyde (SCA), 4-oxo-nonenal (ONE), and isolevuglandins (IsoLGs).

These lipid dicarbonyls modify lysine residues of proteins. While MDA was shown to play a role in MPO-mediated PON1 inactivation (26), whether other lipid dicarbonyls produced downstream of MPO association with HDL can also contribute to PON1 inactivation is unknown. 4-Ketoaldehydes such as IsoLGs are far more reactive with lysine than MDA and rapidly form reversible Schiff base adducts that then mature into irreversible pyrrole and oxidized pyrrole adducts on the lysyl residues of protein (Fig. 1). *Mpo*^*−/−*^ mice have lower levels of IsoLG–protein adducts than WT mice (27). *In vitro* incubation of MPO with HDL increases HDL modification by both MDA and IsoLGs (26, 28). HDL modification by IsoLGs is increased in patients with familial hypercholesterolemia (FH) (28), a group with highly enhanced risk for cardiovascular disease. Dicarbonyl scavengers such as 2-hydroxylbenzylamine and 5-O′-pentyl-pyridoxamine block the modification of lysines by IsoLGs, ONE, SCA, and MDA, with these scavengers having greater reactivity for IsoLG than MDA (29, 30). We have shown that treatment of *Ldlr−/−* mice with these scavengers not only reduces their HDL modification by both IsoLG and MDA (31) but also enhances their PON1 activity (26) and reduces their atherosclerosis (31). We therefore used *in vitro* studies to test the hypothesis that IsoLG or other 4-ketoaldehyde modification of PON1 contributed to MPO-mediated inactivation of PON1.

**Figure 1:**
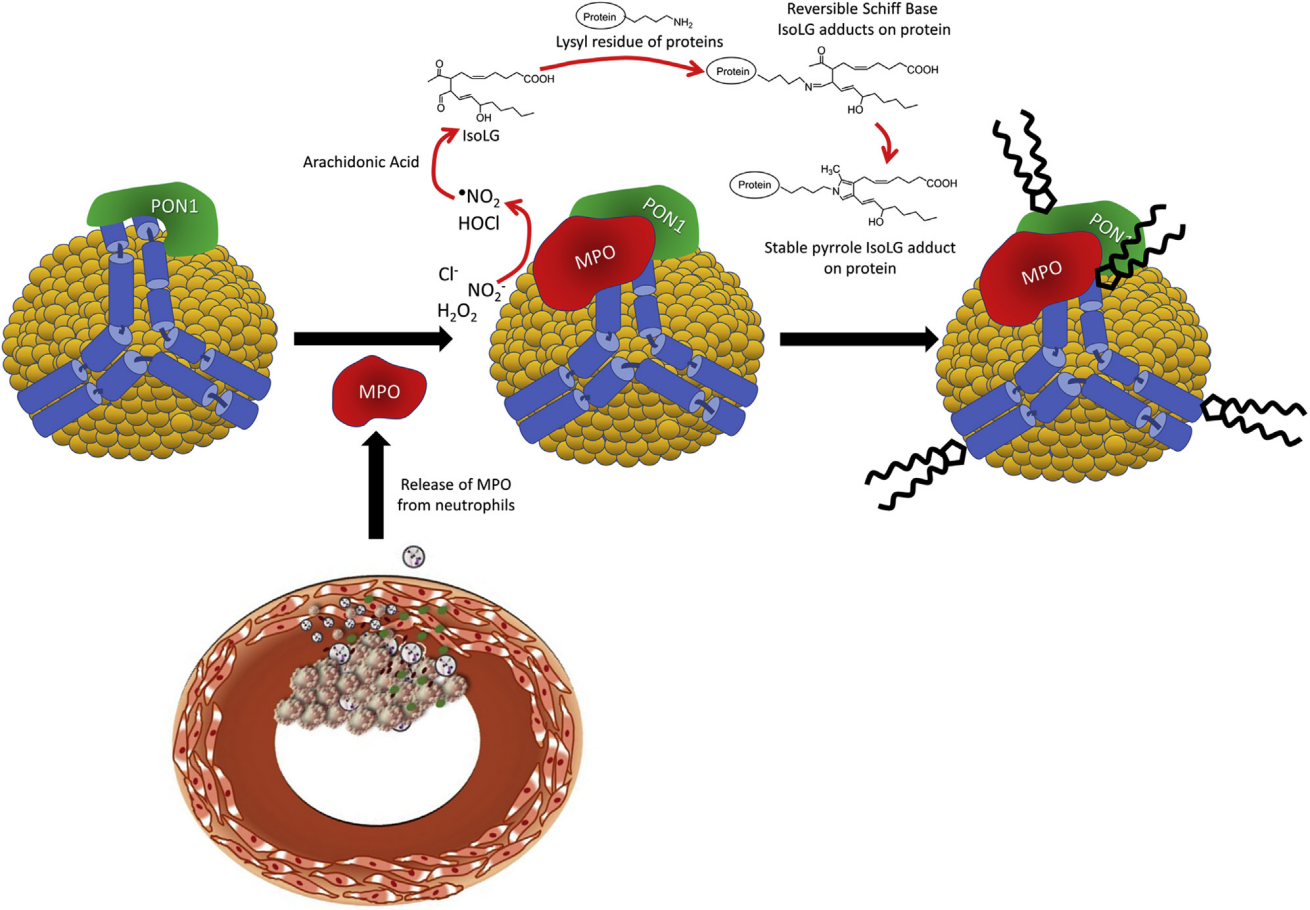
**Schematic of PON1–MPO interaction with apoA-I on HDL and the production of IsoLG.** Activated neutrophils at the site of atherosclerotic lesions release MPO, which associates with circulating HDL. MPO forms a ternary complex with apoA-I and PON1. MPO catalyzes the formation of reactive oxygen species such as hypochlorite, which peroxidizes arachidonic acid to form IsoLG. IsoLG reacts extremely rapidly with primary amines such as the lysyl residues of HDL proteins such as apoA-I to form covalent adducts. HDL, high-density lipoprotein; IsoLGs, isolevuglandins; MPO, myeloperoxidase; PON1, paraoxonase 1.

## Results

### IsoLG inhibits plasma PON1 lactonase activity to a greater extent than other lipid 4-ketoaldehydes

Lipid peroxidation generates several species of highly reactive 4-ketoaldehydes including IsoLG, SCA, and ONE, all of which rapidly react with lysines of HDL proteins, but which differentially exert inhibitory effects on HDL functions such as cholesterol efflux and anti-inflammation (28, 32). To compare the potency each of these 4-ketoaldehydes on PON1, we treated human plasma with up to 20 μM of each aldehyde and examined the effect on PON1 lactonase activity.

We chose to analyze PON1 lactonase activity (by hydrolysis of 5-thiobutyl butyrolactone [TBBL]) (33) rather than PON1 arylesterase and phosphotriesterase activity because lactonase activity was reported to more accurately reflect serum PON1 activity associated with HDL than paraoxonase or arylesterase activity (34). Reductions in PON1 lactonase activity have been shown to be better biomarkers of cardiovascular disease than reductions in arylesterase and phosphotriesterase activity (11).

**Figure 2:**
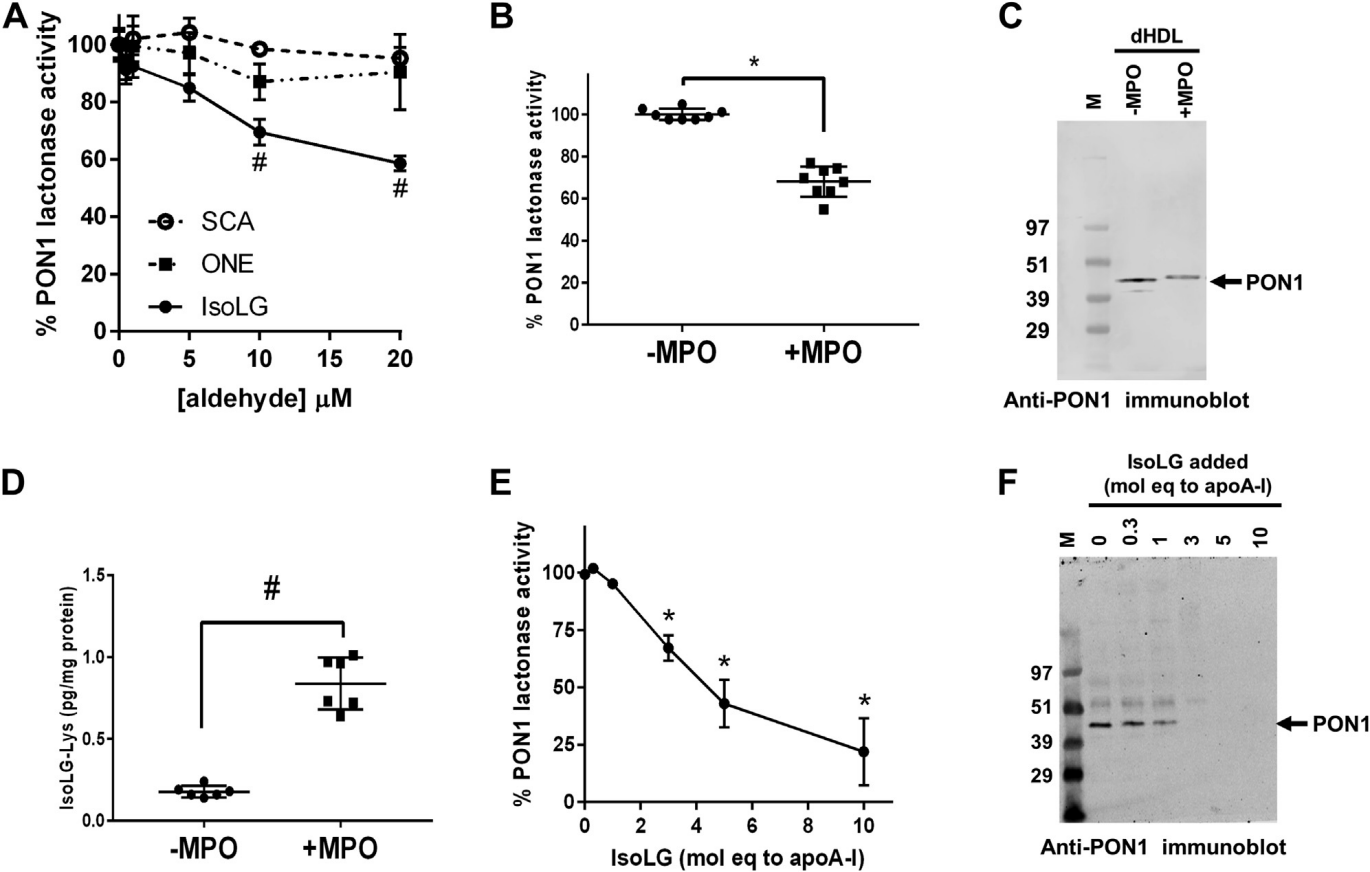
**Exposure of dHDL to MPO alters PON1 activity and mobility, and this effect is mimicked by IsoLG.***A*, diluted human plasma was incubated with succinaldehyde (SCA, n = 4 replicates per concentration), 4-oxo-nonenal (ONE, n = 6), or isolevuglandin (IsoLG, n = 6) for 45 min before initiation of lactonase assay by addition of DTNB and TBBL. IsoLG significantly inhibited PON1 lactonase activity; one-way ANOVA; *p* ≤ 0.0001; ∗*p* < 0.001 for Dunnett’s multiple comparisons test *versus* 0 IsoLG. SCA and ONE failed to inhibit plasma PON1 lactonase activity (one-way ANOVA; *p* = 0.8373 and *p* = 0.8294, respectively). *B*, dextran-isolated HDL (dHDL) was incubated with (+MPO) or without myeloperoxidase (−MPO) for 24 h, and PON1 lactonase activity was measured by hydrolysis of TBBL, ∗*p* = 0.001, unpaired *t* test with Welch correction. *C*, effects of MPO incubation with dHDL for 24 h on molecular weight and crosslinking were measured using SDS-PAGE followed by immunoblotting with the anti-PON1 antibody. A representative immunoblot is shown from two separate experiments. Relative density of +MPO band was 51.0% and 50.3% of the −MPO band for the two experiments. *D*, total IsoLG–Lys adducts present in dHDL preparations incubated with MPO for 24 h were measured by LC/MS; ^#^*p* = 0.0001, unpaired *t* test with Welch correction. *E*, IsoLG was added to dHDL at 0 to 10 mol eq of IsoLG per mole of apoA-I in the dHDL preparation and PON1 lactonase activity measured by hydrolysis of TBBL, mean and range, for two replicates; one-way ANOVA; ∗*p* < 0.05 for Dunnett’s multiple comparisons test *versus* 0 IsoLG. *F*, effects of IsoLG addition to dHDL on molecular weight and crosslinking were measured using SDS-PAGE followed by immunoblotting with the anti-PON1 antibody. DTNB, 5,5′-dithiobis(2-nitrobenzoic acid); IsoLGs, isolevuglandins; M, molecular weight markers; MPO, myeloperoxidase; PON1, paraoxonase 1; TBBL, 5-thiobutyl butyrolactone.

Treatment with IsoLG, but not SCA or ONE, significantly reduced plasma lactonase activity (Fig. 2*A*). This result suggests that not all lipid dicarbonyls produced by lipid peroxidation modify HDL in a manner that alters PON1 activity but that IsoLG modification does. For this reason, subsequent studies focused on the potential role of IsoLG in mediating MPO-induced reductions in PON1 activity.

### Association of MPO with dextran-isolated HDL results in protein modification by IsoLG

To confirm the effect of MPO on the PON1 activity of HDL, purified MPO was incubated with human plasma HDL that had been isolated by centrifugation in dextran solution. Incubation of dextran-isolated HDL (dHDL) with MPO reduced its PON1 lactonase activity by approximately 30% (Fig. 2*B*). Furthermore, incubation of dHDL with MPO slowed the mobility of PON1 in SDS-PAGE (Fig. 2*C*), suggesting that PON1 had undergone post-translational modification. Because incubation of MPO with HDL has previously been shown to generate IsoLG adducts, we measured the extent to which HDL lysines underwent IsoLG modification during incubation of dHDL with MPO. Under these conditions, IsoLG-Lys adducts increased about 3-fold (Fig. 2*D*).

### IsoLG modification of dHDL mimics the effect of MPO association with HDL

We hypothesized that the effects of MPO on PON1 activity and altered mobility in SDS-PAGE involved generation of IsoLG that modified HDL proteins including PON1. To test whether modification of HDL with IsoLG was sufficient to recapitulate the effects of MPO, we added varying concentrations of synthetic IsoLG to dHDL and measured PON1 lactonase activity. IsoLG inhibited PON1 lactonase activity with an IC_50_ of approximately 4 molar equivalents (mol eq) relative to apoA-I (Fig. 2*E*). IsoLG treatment of dHDL also slowed PON1 mobility on SDS-PAGE and decreased the immunoreactivity of the PON1 antibody during immunoblotting (Fig. 2*F*). These findings are consistent with IsoLG modifying PON1 and altering its antigenic sites and enzymatic activity.

### Density gradient ultracentrifugation-isolated HDL lacks PON1 activity but enhances activity of recombinant PON1

**Figure 3:**
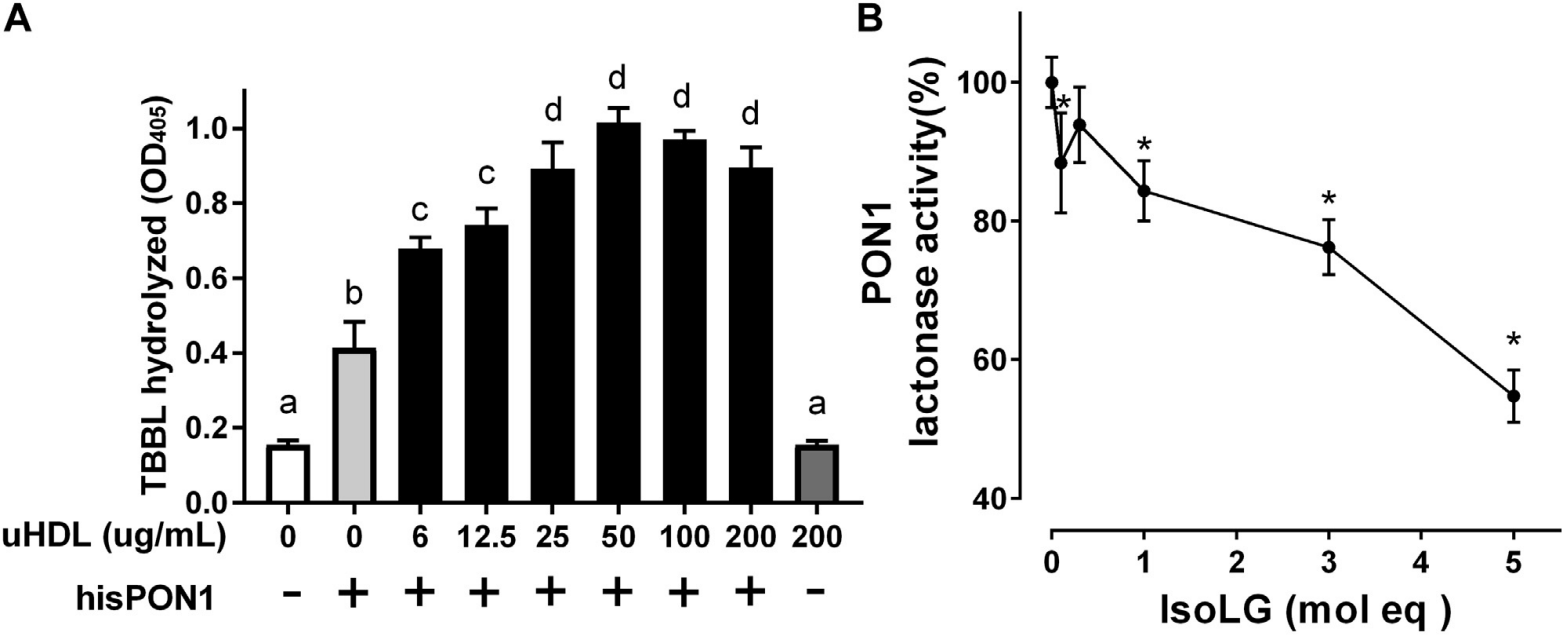
**uHDL enhances the lactonase activity of PON1 which is inhibited by IsoLG.***A*, hisPON1 (0.1 μg) was incubated with varying concentrations (6–200 μg/ml) of density gradient uHDL and then lactonase activity measured by TBBL hydrolysis measured at *A*_405 nm_ (n = 3 replicates, mean ± SD). 200 μg/ml uHDL without any bound hisPON1 failed to hydrolyze TBBL to any greater extent than buffer alone. One-way ANOVA; *p* < 0.0001; Tukey's multiple comparisons test, groups with the same letter designation are not statistically different from each other. *B*, addition of IsoLG (0–5 apoA-I mol eq) inhibited the lactonase activity of hisPON1–uHDL particles (0.2 μg hisPON1/20 μg uHDL). (n = 3 replicates, mean ± SD). One-way ANOVA, *p* < 0.0001, Tukey's multiple comparisons test; ∗*p* < 0.05 *versus* 0 IsoLG. IsoLGs, isolevuglandins; PON1, paraoxonase 1; TBBL, 5-thiobutyl butyrolactone; uHDL, ultracentrifugation-isolated HDL.

Coomassie Blue staining of dHDL showed that this method of HDL preparation retained considerable amounts of non-HDL proteins including albumin (Fig. S1*A*). We considered the possibility that these extraneous proteins increased the amount of IsoLG required to inhibit HDL PON1 activity. We therefore isolated HDL from human plasma by density gradient ultracentrifugation (Fig. S1*B*). Unfortunately, this density gradient ultracentrifugation-isolated HDL (uHDL) showed no PON1 activity despite immunoblotting demonstrating that PON1 was present in uHDL (Fig. S1, *C* and *D*). This is consistent with previous reports that density gradient isolation of HDL inhibits PON1 activity, most likely by stripping out the required calcium and that incubation of uHDL with calcium does not restore PON1 activity (35). Previously, binding of recombinant PON1 to apoA-I in synthetic HDL was shown to markedly increase PON1 activity, particularly its lactonase activity (7). We tested whether uHDL could still induce a similar effect on PON1. Incubation of 6xHis-tagged recombinant human PON1 (hisPON1) with uHDL increased hisPON1 lactonase activity about 3-fold (Fig. 3*A*). Treatment of hisPON1 bound to uHDL (hisPON1–uHDL) with synthetic IsoLG resulted in reduced PON1 activity, similar as seen for IsoLG treatment of dHDL (Fig. 3*B*).

### IsoLG modification inhibits PON1 antilipid peroxidation activity

**Figure 4:**
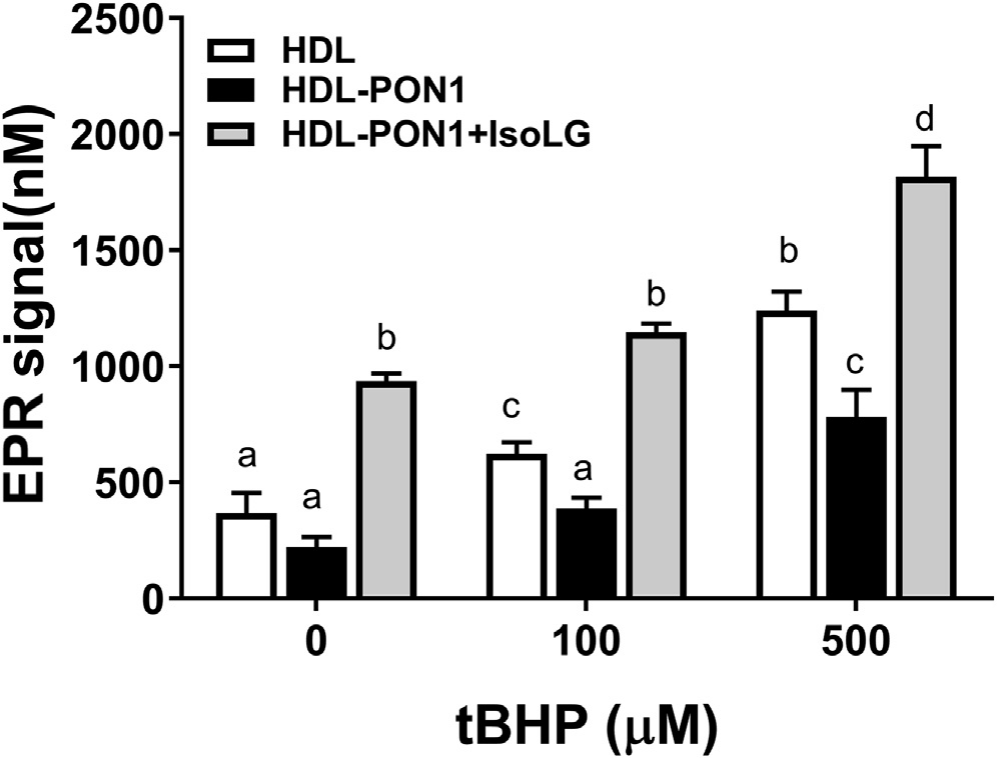
**IsoLG modification of hisPON1–uHDL ablates its antiperoxidation activity.** HisPON1 preincubated with density gradient ultracentrifugation HDL (hisPON1–uHDL) was incubated with or without 1.6 mol eq IsoLG for 24 h. uHDL only was used as a negative control. Samples were treated with 0 μM, 100 μM, and 500 μM tert-butyl hydroperoxide (tBHP), and lipid peroxidation was measured by EPR using T10M-H as the spin probe. Each data point represents peak signal intensity from EPR spectra (n = 3). Two-way ANOVA, *p* < 0.0001 for tBHP concentration, F(2,18) = 231.6; *p* < 0.0001 for HDL composition type, F(2,18) =264.9; *p* = 0.102 for interaction, F(4,18) = 4.559. Tukey's multiple comparisons test: groups with the same letter designation are not statistically different from each other. EPR, electron paramagnetic resonance; HDL, high-density lipoprotein; hisPON1, 6xHis-tagged recombinant human PON1; IsoLGs, isolevuglandins; uHDL, ultracentrifugation-isolated HDL.

Like its lactonase activity, PON1’s antilipid peroxidation activity appears critical to its antiatherosclerotic effects. To measure PON1 antilipid peroxidation activity, we developed an assay that used tert-butyl-hydroperoxide (tBHP) to initiate lipid peroxyl radical formation within HDL preparations, and then, the spin probe 4-decyloxy-1-hydroxy-2,2,6,6-tetramethylpiperidine hydrochloride (TM10-H) was used to trap and measure these lipid peroxyl radicals by electron paramagnetic resonance (EPR) (Supporting information). We used this antilipid peroxidation assay to examine the effect of IsoLG modification (1.6 mol eq) on the ability of hisPON1–uHDL to control levels of lipid peroxyl radicals. As a control, we used uHDL without added hisPON1. Addition of hisPON1 to uHDL markedly reduced lipid peroxyl radical levels when uHDL preparations were exposed to tBHP (Fig. 4 and Fig. S2). IsoLG modification of hisPON1–uHDL not only eliminated the protective effect of hisPON1 but also further enhanced lipid peroxyl radical levels. Therefore, IsoLG modification of HDL inhibits both PON1 lactonase and antiperoxidation activities.

### IsoLG modification of HDL inhibits PON1 activity by direct modification of PON1

**Figure 5:**
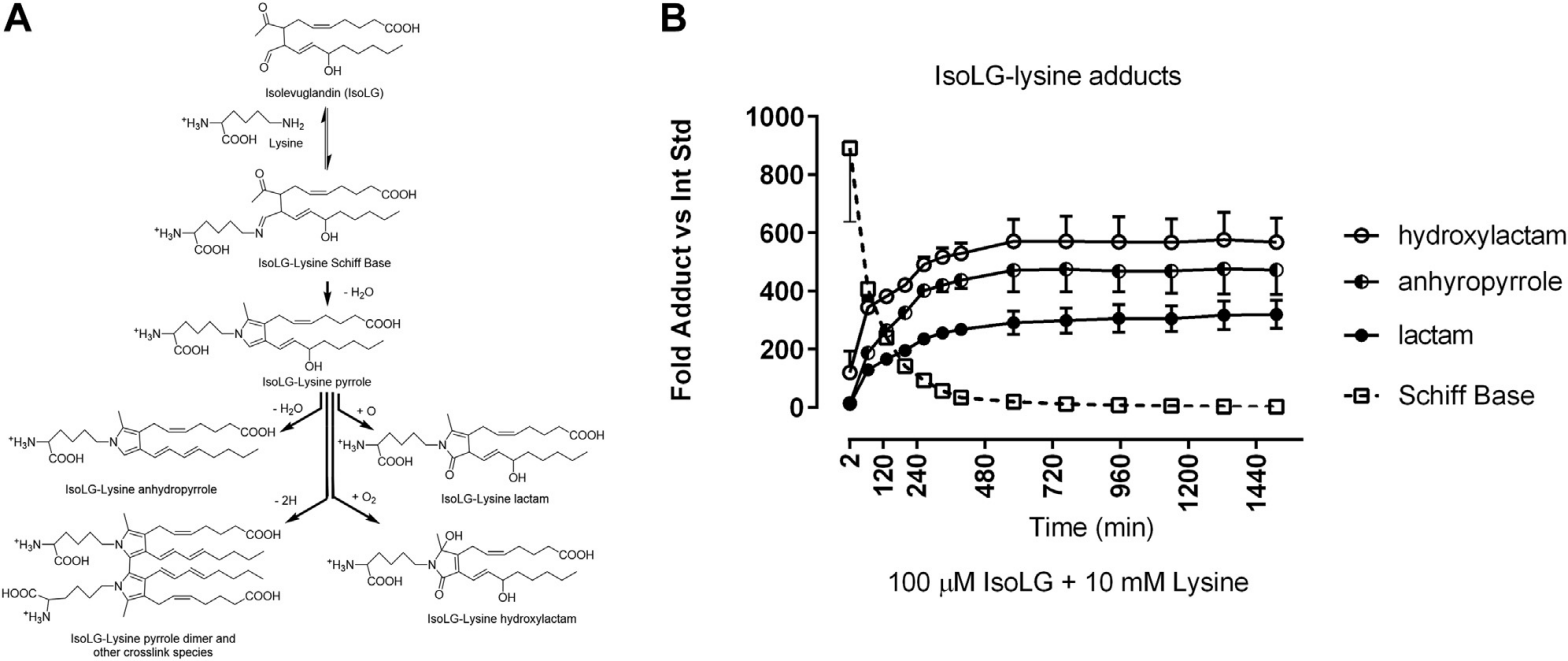
**Time course for formation of stable IsoLG–lysine pyrrole adducts.***A*, schematic of IsoLG reaction with lysine to initially form highly reversible Schiff base adducts, followed by the formation of irreversible pyrrole adducts and their derivatives including anhydropyrrole adduct (by loss of water), lactam adduct (by addition of single oxygen atom), and hydroxylactam adduct (by addition of molecular oxygen). *B*, abundance of each adduct species (measured by LC/MS relative to an internal standard) at each time point after addition of 10 mM lysine to 100 μM IsoLG. IsoLGs, isolevuglandins.

We next investigated the mechanism whereby IsoLG inhibited PON1 activity associated with HDL. Because IsoLG modifies lysyl residues, we hypothesized that IsoLG modification of PON1 lysines would inhibit its activity. However, there are far more lysines available for modification on apoA-I than there are on PON1. Furthermore, only about one in every ten HDL particles carry a molecule of PON1 (36), whereas essentially every HDL particle carries at least one apoA-I. In this context, our finding that IsoLG modification of HDL potently inhibited PON1 activity is somewhat surprising; however, even if all the initial reaction of IsoLG was with apoA-I, it might still inhibit PON1 in several ways. First, the initial modification of lysyl residues by IsoLG forms Schiff base adducts that are reversible. For this reason, some of the IsoLG that had initially reacted with apoA-I could undergo the reverse reaction to free aldehyde that could then undergo reaction with PON1. Second, Schiff Base adducts can cyclize to form irreversible pyrrole adducts, and these pyrrole adducts can undergo additional reactions including formation of oxidized pyrrole monoadducts such as lactam and hydroxylactam adducts, as well as pyrrole–pyrrole crosslinks (Fig. 5*A*). Formation of crosslinks between apoA-I and PON1 could cause loss of PON1 activity. Third, formation of stable oxidized pyrrole adducts on apoA-I could inhibit apoA-I’s ability to hyperactivate PON1. In the absence of this apoA-I-mediated hyperactivation, overall PON1 activity would be reduced more than 60%, essentially the same as inhibiting the enzyme directly.

To determine the time course for formation of various IsoLG–lysine adducts, IsoLG was reacted with excess lysine for up to 24 h, and the levels of each monoadduct formed were monitored by LC/MS (Fig. 5*B*). Levels of crosslink adducts were not monitored because of the large number of possible species (37). Maximal levels of Schiff base adducts formed within 2 min of starting the reaction. Conversion of these Schiff base adducts to other monoadducts followed rapidly, so that by 2 h, about half of the Schiff base adducts had been converted to other monoadducts, and within 12 h, all Schiff base adducts appeared to have been converted to various forms of pyrrole adducts. These results suggested that all three mechanisms involving an initial reaction of IsoLG with apoA-I are possible.

**Figure 6:**
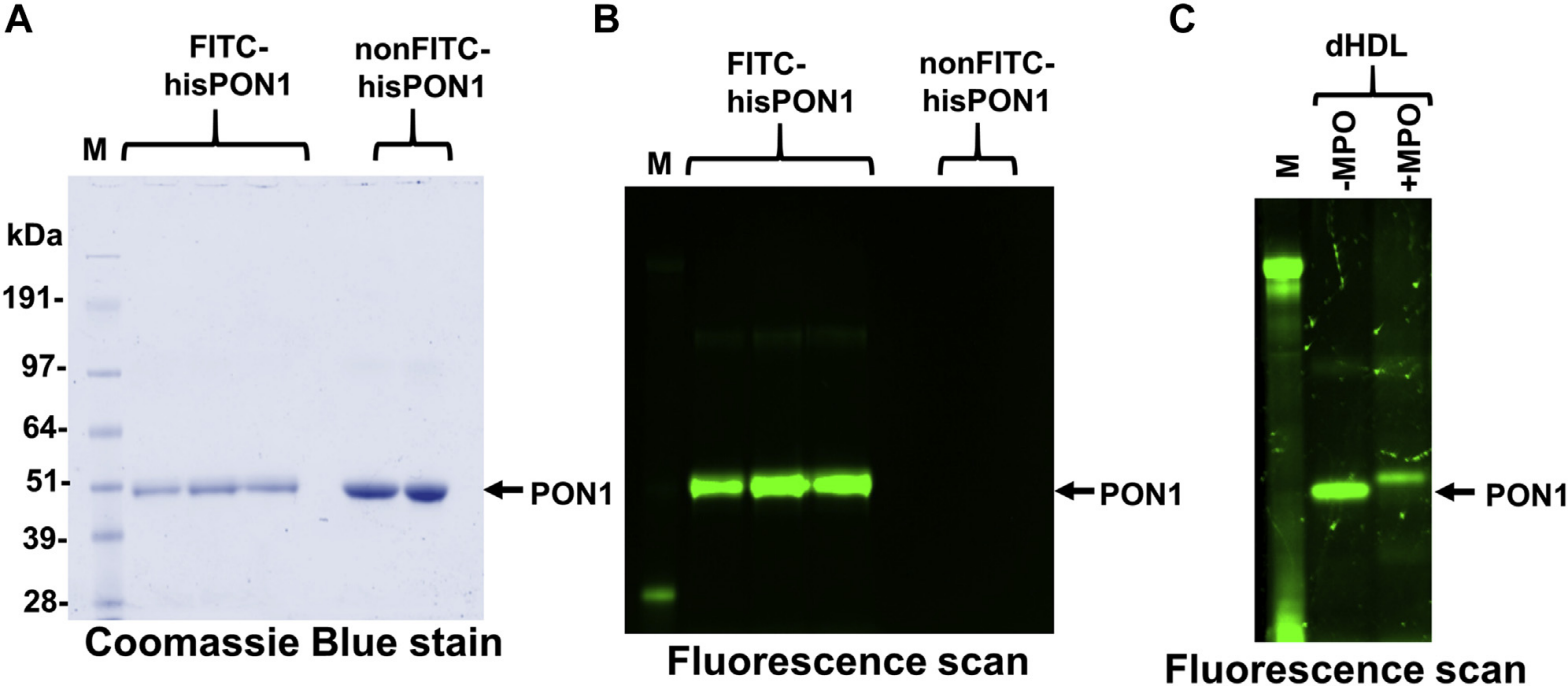
**FITC-labeled hisPON1 is not crosslinked to apoA-I after incubation with MPO.** Replicate samples of FITC-hisPON1 (triplicate) and non–FITC-hisPON1 (duplicate) were run on SDS-PAGE, and the membrane visualized by (*A*) Coomassie Blue staining and (*B*) fluorescence imaging (ex488/em520 nm). *C*, FITC-hisPON1 was then preincubated with dextran-isolated HDL (dHDL) for 1 h and then incubated with or without MPO for 24 h before SDS-PAGE analysis with fluorescence imaging. HDL, high-density lipoprotein; MPO, myeloperoxidase; PON1, paraoxonase 1.

Incubation of HDL with either MPO or synthetic IsoLG markedly increases apoA-I crosslinking (28). For this reason, we considered MPO-induced IsoLG crosslinking of apoA-I to PON1 to be possible although the PON1 immunoblot studies of HDL treated with MPO or IsoLG (Fig. 2) did not show immunoreactive bands at ∼70 kD as would be expected for a PON1- apoA-I crosslink. One potential explanation for the lack of such an immunoreactive band is that the PON1 antibody might be unable to recognize PON1 when crosslinked by IsoLG to apoA-I. To detect PON1 without relying on immunoreactivity, we fluorescently labeled hisPON1 using FITC and added this FITC-hisPON1 to dHDL. Incubation of FITC-hisPON1–dHDL with MPO produced a slight upward shift of FITC-hisPON1 on SDS-PAGE as previously seen with immunoblotting, but no fluorescence in the ∼70 kDa region (Fig. 6). These results suggest that PON1 inhibition is not the result of IsoLG crosslinking apoA-I to PON1.

**Figure 7:**
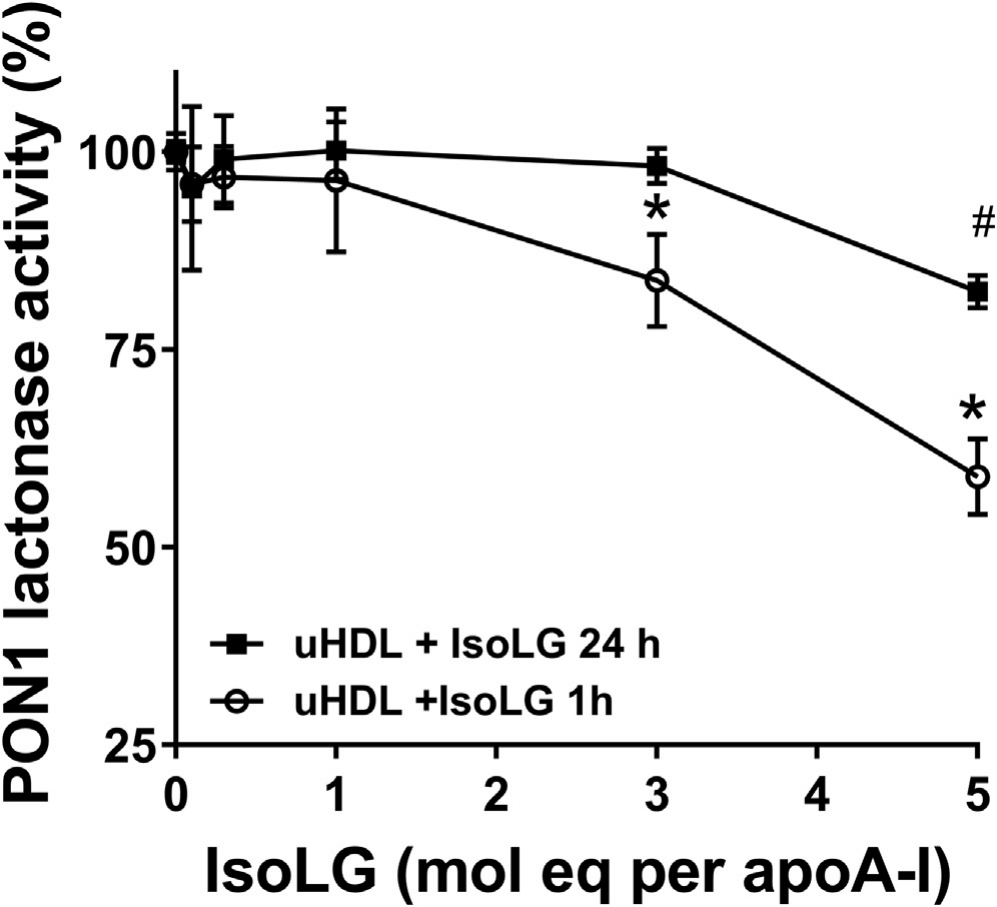
**Effects of stable *versus* reversible IsoLG adducts on the capacity of uHDL to induce PON1 activity.** Density gradient ultracentrifugation-isolated HDL (uHDL) was modified with 0.1 to 5 mol eq IsoLG (relative to apoA-I) for either 1 h (reversible adducts) or 24 h (stable adducts). HisPON1 was then incubated with modified uHDL for 1 h, and PON1 lactonase activity was measured by TBBL hydrolysis. Two-way ANOVA; *p* < 0.0001, F(5,78) = 60.71 for IsoLG concentration; *p* < 0.0001, F(1,78) = 39.11 for treatment time; *p* < 0.0001 for interaction, F(5,78) = 11.55; ∗*p* < 0.05 *versus* no IsoLG 1 h; ^#^*p* < 0.05 *versus* no IsoLG 24 h. hisPON1, 6xHis-tagged recombinant human PON1; IsoLGs, isolevuglandins; PON1, paraoxonase 1; TBBL, 5-thiobutyl butyrolactone.

To test if IsoLG that initially reacted with apoA-I was available to modify PON1 or if irreversible IsoLG adducts on apoA-I prevented it from hyperactivating PON1, various concentrations of IsoLG (0.1–5 mol eq) were reacted with uHDL for 1 h or 24 h to generate reversible or irreversible IsoLG adducts, respectively. HisPON1 was incubated with the modified uHDL and its lactonase activity measured (Fig. 7). uHDL irreversibly modified with 0.1 to 3 mol eq IsoLG activated PON1 to a similar extent as unmodified uHDL, and uHDL irreversibly modified with 5 mol eq IsoLG still activated PON1 to 82% of normal. These results suggest that irreversible IsoLG modification of apoA-I had little impact on the ability of apoA-I to hyperactivate PON1. In contrast, uHDL reversibly modified with 5 mol eq IsoLG before addition of PON1 could only activate PON1 to 59% of its normal PON1 activity. These results suggested that reversible modification of apoA-I released free IsoLG that could subsequently modify closely adjacent PON1.

To measure available IsoLG, we used 10 kDa molecular weight cut-off filters to separate protein from released IsoLG and then reacted the filtrate with excess lysine for 24 h and quantified the resulting IsoLG–Lys adducts by LC/MS. Significant amounts of lysine adducts were detected in the filtrate from the 1-h IsoLG + uHDL preparation, but negligible adducts were detected in the filtrate from the 24-h IsoLG + uHDL preparation (Fig. S3). These results suggest that the reduction in PON1 activity seen in the 1-h IsoLG + uHDL preparation is most likely the result of IsoLG reacting with PON1 rather than *via* modification of apoA-I that prevents PON1 hyperactivation.

**Figure 8:**
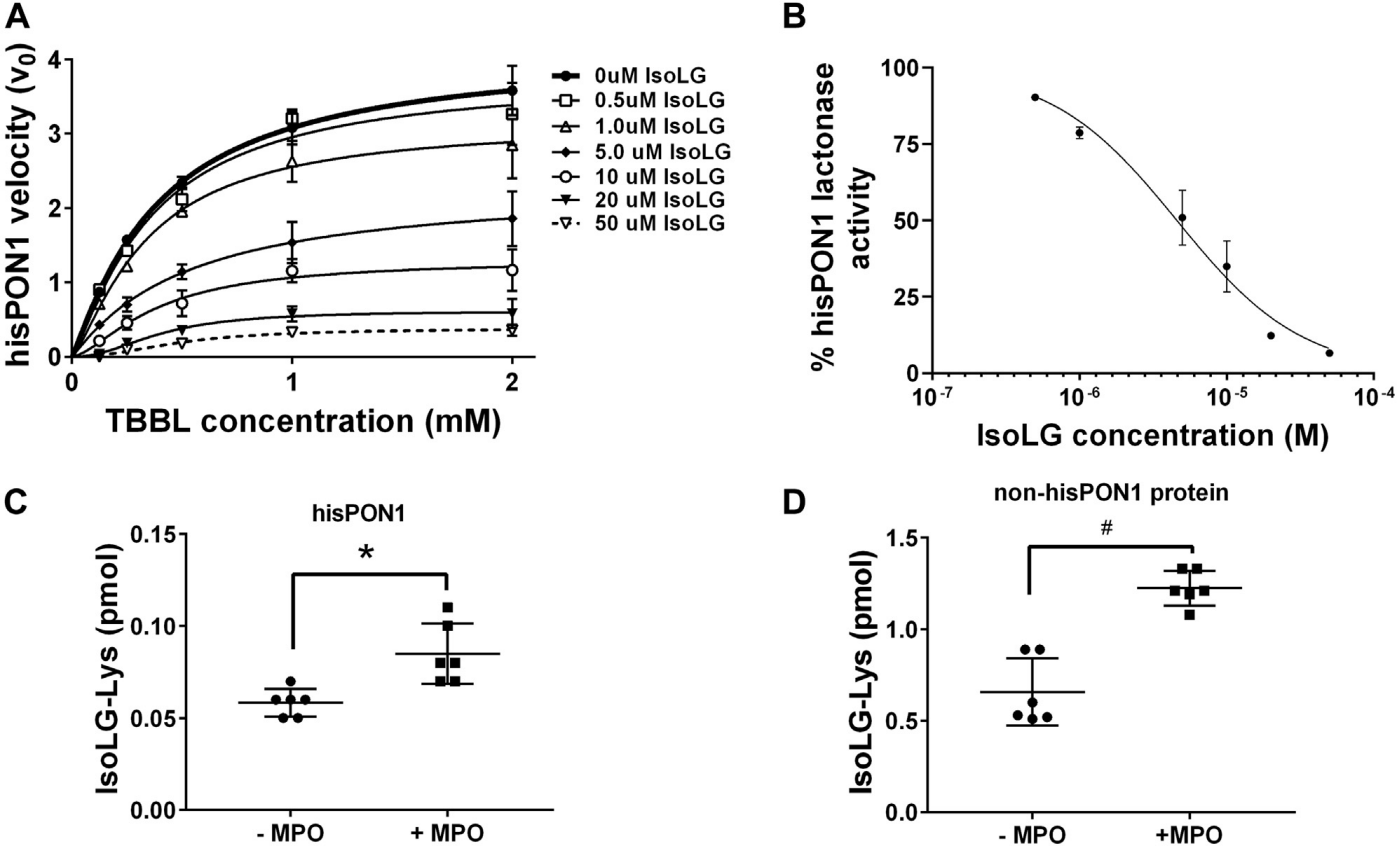
**Direct addition of IsoLG to hisPON1 results in inhibition of PON1 activity, and incubation of MPO with hisPON1–uHDL induces IsoLG modification of PON1.***A*, effect of IsoLG and TBBL concentration on initial velocity of PON1 lactonase activity (mean ± SEM, n = 3). Concentration response curves were performed using 0 to 50 μM IsoLG and 0.125 to 2.0 mM TBBL. HisPON1 (0.0075 μM) was incubated with the indicated concentrations of IsoLG for 45 min and then TBBL hydrolysis measured at 412 nm for 45 min. *B*, normalized concentration response curve for IsoLG with TBBL concentration 0.25 mM. All initial velocities were normalized to an average for 0 IsoLG. *C*, preparations of hisPON1 preincubated with density gradient ultracentrifugation-isolated HDL (hisPON1-uHDL) were incubated with (+MPO) or without myeloperoxidase (−MPO) for 24 h and then affinity-purified using nickel beads. Levels of the IsoLG protein adduct (IsoLG–Lys) were measured in the affinity purification fraction representing isolated hisPON1 (n = 6), unpaired two-tailed *t* test, ∗*p* = 0.005. *D*, the supernatant fraction representing all other uHDL proteins of hisPON1–uHDL (non–hisPON1 proteins) (n = 6); unpaired two-tailed *t* test, ^#^*p* < 0.0001. hisPON1, 6xHis-tagged recombinant human PON1; IsoLGs, isolevuglandins; MPO, myeloperoxidase; PON1, paraoxonase 1; TBBL, 5-thiobutyl butyrolactone.

To examine if direct modification of PON1 by IsoLG results in inactivation, we incubated hisPON1 with varying concentrations of IsoLG and then added varying amounts of TBBL to assess changes in *K*_m_ and *V*_max_. Increasing the concentration of IsoLG markedly reduced *V*_max_ while not altering *K*_m_, consistent with noncompetitive inhibition (Fig. 8*A*). The concentration response curve obtained for 0.25 mM TBBL gave an IC_50_ for IsoLG of 4.6 μM (Fig. 8*B*).

### MPO association with HDL results in IsoLG modification of PON1

Our findings suggest that direct modification of PON1 is the most likely mechanism by which IsoLG produced by MPO-catalyzed peroxidation of HDL induced inhibition of PON1 activity. We therefore sought to determine if PON1 was modified by IsoLG after exposure of hisPON1-uHDL to MPO. HisPON1 was incubated with uHDL for 1 h and then MPO added for 24 h. HisPON1 was then isolated away from other HDL proteins using nickel beads.

Initially, we used 0.1% NP-40 detergent in PBS to remove proteins such as apoA-I that might be bound to hisPON1 from the beads. Under these conditions, the amount of IsoLG–protein adducts found associated with the nickel beads was about 3-fold higher for uHDL complexed with both hisPON1 and MPO than uHDL complexed with only MPO or only hisPON1 (Fig. S4*A*). IsoLG–protein adducts not associated with the beads were similar for uHDL complexed with MPO only and uHDL complexed with both MPO and hisPON1 (Fig. S4*B*). However, these conditions were not sufficiently stringent to completely remove all apoA-I associated with the hisPON1–nickel bead complex (Fig. S4, *C* and *D*). Optimization experiments showed that addition of 0.5 M NaCl to 0.1% NP-40 in PBS was sufficient to remove apoA-I without significantly affecting the amount of hisPON1 captured on the nickel beads (Fig. S5).

Using these conditions to remove hisPON1 from other uHDL-associated proteins, we found that treatment with MPO significantly increased IsoLG adducts associated with hisPON1 (Fig. 8*C*). MPO treatment also increased IsoLG adducts in the supernatant, which represents the remaining uHDL proteins including apoA-I (Fig. 8*D*). Based on total IsoLG adducts measured for both nickel beads and supernatant, the increase in IsoLG–PON1 adducts after MPO treatment appears to represent about 4.5% of the total new IsoLG protein adducts formed on uHDL after MPO treatment. HisPON1 constitutes 5.5% of uHDL protein by weight under these conditions, suggesting its modification is almost directly proportionate to its abundance.

## Discussion

Individuals with atherosclerotic cardiovascular disease consistently show reduced circulating PON1 activity (11, 12, 13, 38, 39). Association of MPO with HDL has previously been shown to reduce the PON1 activity of HDL (21, 26) and to correlate with increased atherosclerosis (40). Our studies demonstrate that association of MPO with HDL *in vitro* generates IsoLG that directly modifies the lysine residues of PON1 and that direct modification of PON1 is the primary mechanism by which IsoLG reduces PON1 activity.

Reduced PON1 activity is not simply a marker of atherosclerotic cardiovascular disease but appears to directly promote disease. Ablating PON1 in atherosclerosis-prone mice enhances development of atherosclerosis (9). We therefore sought to identify mechanisms that can lead to reduced PON1 activity. The studies reported here show that modification of PON1 with exogenously added IsoLG markedly reduces PON1 lactonase and antiperoxidation activity. That endogenous modification of PON1 by lipid dicarbonyls including IsoLG and MDA contributes to reduced PON1 activity observed *in vivo* during atherosclerosis is supported by our previous observation that IsoLG modification of HDL is enhanced in patients with FH (28) and *Ldlr*^*−/−*^ mice and that treating *Ldlr*^*−/−*^ mice with dicarbonyl scavengers increased their PON1 activity (26) and reduced atherosclerosis (31).

That MPO impacts PON1 activity *in vivo* was previously demonstrated using i.p. injection of zymosan to induce peritonitis. Zymosan injection reduced PON1 activity by 30% in WT mice and by 55% in transgenic mice overexpressing human MPO, but this injection had no effect on PON1 activity in *Mpo*^*−/−*^ mice (21). Elevated levels of MPO are observed in plasma and serum from individuals with acute coronary syndromes or at risk for major adverse cardiac events (40). MPO bound to HDL can be detected in circulation and atherosclerotic plaques (41, 42, 43). MPO associates with HDL through interactions with apoA-I and PON1 (21), and association of MPO with HDL *in vitro* reduces PON1 activity (21, 26).

Two potential mechanisms by which MPO could inhibit PON1 activity have previously been demonstrated, namely tyrosine modification by hypochlorous acid and ^●^NO_2_ (21) and lysine modification by MDA (26). However, given the robust formation of IsoLG induced by MPO-generated ^●^NO_2_ and the greater reactivity of IsoLG than MDA with lysines, we sought to test whether IsoLG or other 4-ketoaldehydes such as ONE and SCA might also contribute to MPO-mediated inactivation of PON1.

Our interest in the effects of IsoLG on PON1 activity were driven in part by our previous finding that dicarbonyl scavengers (which scavenge MDA but even more effectively scavenge IsoLG, ONE, and SCA) significantly enhanced PON1 activity in *Ldlr*^*−/−*^ mice (26). When we tested three 4-ketoaldehydes for their ability to inhibit plasma PON1 lactonase activity, only IsoLG showed a significant effect. Importantly, incubation of MPO with HDL significantly increased IsoLG–Lys adducts on PON1. That such adducts could inhibit PON1 was shown by our finding that adding IsoLG directly to PON1 inhibited PON1 lactonase activity with 10 mol equivalent of IsoLG being sufficient to inhibit 80% of PON1 activity. Previous studies found that 40 mol eq of MDA is needed to achieve similar inhibition of PON1 activity (26), consistent with the greater reactivity of IsoLG and its greater capacity to disrupt protein structure compared with MDA.

Our studies with IsoLG focused exclusively on PON1 lactonase and antiperoxidation activities. Multiple substrates have previously been used to measure PON1 activity and its relationship to disease (3, 5, 11, 12, 13, 14, 15, 16, 44, 45). In addition to its lactonase and antiperoxidation activity, PON1 exerts arylesterase activity (*e.g.*, hydrolysis of phenyl acetate, 4-chloromethyl-phenylacetate, or 4-naphtyl-acetate) and phosphotriesterase activity (*e.g.*, hydrolysis of paraoxon). Structure–activity relationship studies and directed evolution studies support the notion that the lactonase activity is PON1’s primary native activity (2, 3, 4, 5, 6, 7). Furthermore, lactonase activity appears to be a better biomarker of cardiovascular disease than arylesterase and phosphotriesterase activity (11). That PON1’s antiatherosclerotic activities are primarily due to its lactonase, and antiperoxidation activity is further supported by studies with PON3, which lacks paraoxonase and arylesterase activity while retaining similar lactonase and antiperoxidation activity to PON1 (46). Transgenic overexpression of human PON3 appears to protect *Apoe*^*−/−*^ mice from atherosclerosis to a similar extent as transgenic overexpression of human PON1 (47).

Although we did not study the effect of IsoLG on PON1’s arylesterase and paraoxonase activity, we would anticipate that they would be similarly inhibited because of the active site overlap. PON1’s active site consists of a deep hydrophobic pocket ringed with a number of His residues and with solvent-exposed calcium at its bottom (4). Site-directed mutagenesis and pH-rate profile studies show that His115 and His134 are essential for PON1 lactonase and esterase activity, with smaller contributions from His285 and His184 (4). Phosphotriesterase activity is predominately dependent on His285 and His184 (4). Given shared catalytic residues, it is not surprising that reduced PON1 activity measured using all four substrate activities is associated with cardiovascular disease (3, 11, 12, 13, 14, 15, 16).

Our studies suggest that the primary mechanism for IsoLG modification of HDL to inhibit PON1 is by direct modification of PON1 rather than by modification of apoA-I that altered its interactions with PON1. Somewhat unexpectedly, we found no evidence that MPO treatment of uHDL-hisPON1 led to crosslinking between apoA-I and PON1 despite their close proximity on the surface of HDL and our previous finding of IsoLG crosslinking apoA-I and apoA-II (28). While incubation of uHDL with IsoLG for 1 h markedly reduced its capacity to hyperactivate subsequently added hisPON1, incubation of uHDL with IsoLG for 24 h (thereby forming stable, irreversible IsoLG adducts) hyperactivated hisPON1 that was subsequently added to nearly the same extent as unmodified uHDL. These results suggest that reversible IsoLG Schiff base adducts on HDL proteins (*e.g.*, apoA-I) can be released to then form adducts on PON1, thereby inactivating it. Indeed, adding IsoLG directly to hisPON1 in the absence of HDL markedly inhibited PON1 lactonase activity. That IsoLG generated when MPO associates with HDL can efficiently modify PON1 is shown by our finding that 4.5% of IsoLG adducts on HDL formed under these conditions are on PON1, appropriately proportionate to that 5.5% of total protein mass that hisPON1 represents.

The studies reported here suggest the need for additional studies to determine the extent to which IsoLG modification of PON1 contributes to reduced PON1 activity *in vivo* and to progression of atherosclerosis. While we have previously shown that HDL isolated under conditions that favor atherosclerosis (*e.g.*, patients with FH and *Ldlr*^*−/−*^ mice) has increased IsoLG modification (28, 31), the methods used by those studies do not identify which HDL proteins are modified. Therefore, studies are needed to determine the specific lysine residues of PON1 modified by IsoLG when added *in vitro*, the effect of these modification on PON1 structure, and whether these same modifications are increased for PON1 isolated from individuals with coronary artery disease. It would also be useful to identify the lysine residues of apoA-I that are modified by IsoLG, as this may give insight as to why stable IsoLG modification of apoA-I does not affect its ability to hyperactivate PON1. Previous studies using deuterium exchange showed that the interface regions of apoA-I with PON1 are apoA-I Leu38-Leu46 (which includes Lys40 and Lys45) and apoA-I Thr202-Arg215 (which includes Lys206 and Lys208) (21). Because the proteins associated with HDL differ by subclass and PON1 concentration and activity differ by HDL subclass (HDL3c > HDL3b > HDL3a > HDL 2a > HDL2b), it might be informative for future studies to determine if individual subclasses differ in their vulnerability to PON1 inhibition by IsoLG. Finally, because we have previously shown that dicarbonyl scavengers can increase PON1 activity in *Ldlr*^*−/−*^ mice, it would be valuable to examine the extent to which treatment with these scavengers also reduced IsoLG (and/or MDA) modification of key PON1 residues.

## Experimental procedures

### Reagents and enzymes

All reagents and enzymes were purchased from MilliporeSigma unless otherwise stated. HisPON1 was expressed and purified as described previously (3, 45). Synthesis of 4-ketoaldehydes was as described previously for IsoLG (48), ONE (49), and SCA (50). The synthesis of TBBL and TM10-H is described in Supporting information.

### Isolation of HDL

HDL was isolated from plasma of healthy volunteers at Vanderbilt University Medical Center, purchased from Interstate Blood Bank, or obtained from the Vanderbilt blood bank. The study conformed to the guidelines set out in the Declaration of Helsinki and pertinent ethical regulations. The protocol was approved by the ethical committee of Vanderbilt University Medical Center (IRB# 151573), and all volunteers gave written informed consent for participation in the study.

dHDL was extracted from human plasma using an HDL purification kit (Cell Biolabs, STA-607). Fifty microliter of dextran solution and 500 μl of precipitation solution was added to 10 ml plasma, mixed, and incubated on ice for 5 min and then centrifuged at 6000*g* at 40 °C for 10 min. The supernatant was collected, mixed with 600 μl of dextran solution and 1500 μl of precipitation solution, and incubated at room temperature (RT) for 2 h. After centrifugation at 6000*g* at 40 °C for 30 min, the pellet was suspended in 5 ml of the resuspension buffer, centrifuged again at 6000*g* at 40 °C for 10 min, and the pellet suspended in 6 ml of 1× HDL wash solution with shaking at 40 °C for 30 min. Nine hundred microliter dextran removal solution was added and then incubated at 40 °C for 1 h. Samples were finally centrifuged at 6000*g* at 40 °C for 10 min, and the supernatant was dialyzed in 1× PBS at 4 °C overnight.

Density gradient uHDL was isolated from human plasma by density gradient ultracentrifugation (1.061–1.21 g/ml), 330,000*g*, followed by extensive dialysis at 4 °C in 1X PBS as we have previously described (28).

### Treatment of plasma with 4-ketoaldehydes

Human plasma was diluted 100-fold in the lactonase activity buffer (10 mM PBS, pH 7.5, 1 mM CaCl_2_) and then incubated with up to 20 μM of IsoLG, ONE, or SCA for 45 min before initiating the lactonase assay by addition of 5,5′-dithiobis(2-nitrobenzoic acid) (DTNB) (0.8 mM final) and TBBL (1 mM final).

### Treatment of dHDL with MPO

dHDL was oxidized with a commercial preparation of MPO (#475911, MilliporeSigma) as described previously (28). Briefly, 25 μg of dHDL (1 mg/ml) was incubated with or without MPO-oxidase system (57 nM MPO, 100 μg/ml glucose, 20 ng/ml glucose-oxidase (49180, MilliporeSigma), and 0.05 mM NaNO_2_) in total 25 μl of the activity buffer containing 10 mM sodium phosphate (pH 7.5), 200 μM diethylenetriaminepentaacetic acid, 1 mM CaCl_2_ at 37 °C overnight. On the next day, half of the sample was used for Western blot analysis carried out using a monoclonal antibody specific for human PON1 and the other half used to measure PON1 lactonase activity. The samples were diluted in the lactonase assay buffer and 10 μl of 10 mM product DTNB (0.8 mM final concentration) and then 10 μl of TBBL added (1 mM final concentration). Lactonase activity was measured as change in absorbance at 405 nm, normalized to activity in absence of MPO.

Quantitation of IsoLG–Lys adducts on dHDL by IsoLG was performed as described previously (28, 51, 52). Briefly, dHDL with or without MPO after overnight incubation was subjected to proteolysis with Pronase and aminopeptidase M. The amount of IsoLG–lysyl–lactam was then measured by stable-isotope dilution LC/MS/MS.

### Effect of synthetic IsoLG on dHDL

Twenty microgram of dHDL (400 μg/ml final) was incubated for 24 h with increasing concentrations of IsoLG (0.3 mol eq of IsoLG/mol of apoA-I to 10 eq of IsoLG/mol of apoA-I) at 37 °C in total 50 μl of lactonase assay buffer. The next day, samples were diluted in 50 μl of the lactonase assay buffer, and lactonase activity was measured using DTNB and TBBL as described above. The extent of protein crosslinking was detected by visualizing PON1 band on Western blot.

### FITC labeling of hisPON1 labeling and determination of MPO effects

FITC labeling of PON1 was then performed as described previously (53). After using dialysis to exchange PON1 into borate buffer (0.1 M borate, 25 mM tetraborate, 150 mM NaCl, 0.1% Tergitol and 2 mM DTT, pH 8.6), 1 mg/ml FITC was prepared in dimethylformamide and 50 μl added dropwise to 1 mg/ml hisPON1 solution and then incubated for 1 h at RT with constant stirring. FITC-hisPON1 solution then passed through Sephadex-25 column pre-equilibrated with the separation buffer (10 mM phosphate buffer, pH 7.5, containing 150 mM NaCl, 0.1% Tergitol and 2 mM DTT), to remove unbound FITC. FITC-hisPON1 was eluted with PBS, concentrated using 3 kDa centrifugal filter, and stored at −20 °C. To test the effect of MPO, 25 μg of dHDL was preincubated with 0.2 μg FITC-hisPON1 for 1 h to form a complex and then further incubated with or without MPO-oxidase system (57 nM MPO, 100 μg/ml glucose, 20 ng/ml glucose-oxidase, and 0.05 mM NaNO_2_) in total 25 μl of the activity buffer containing 10 mM sodium phosphate (pH 7.5), 200 μM diethylenetriaminepentaacetic acid, and 1 mM CaCl_2_ at 37 °C overnight. The next day, samples were analyzed by SDS-PAGE and gel was scanned with Bio-Rad ChemiDoc imager using ex488/em520 nm filter.

### Recombinant hisPON1 activation studies

Purified hisPON1 was reconstituted in 10 mM Hepes, pH 7.5, 1 mM CaCl_2_, 1 mM DTT, and 0.01% Tergitol for activation experiments. For activation experiments, uHDL (6–200 μg/ml) was diluted in the lactonase assay buffer and then 0.1 μg hisPON1 was added. As a negative control, buffer alone, hisPON1 alone, and uHDL without hisPON1 were used. HisPON1 lactonase activity was measured using DTNB and TBBL as described above.

To test if IsoLG affects the hisPON1 activity in uHDL precomplexed with free hisPON1 similar to dHDL, the dose-dependent effect of IsoLG was determined on hisPON1 activation in dHDL precomplexed with free hisPON1. Twenty microliter of 1 mg/ml dHDL (400 μg/ml) was incubated with 0.2 μg hisPON1 in total 50 μl of the lactonase assay buffer for 1 h to form complex and then treated with increasing concentrations of IsoLG (0–5 mol eq to HDL apoA-I) for 1 h. Samples were diluted in 45-μl assay buffer and the percentage enzymatic activity measured with TBBL as described above.

### Effect of IsoLG on antiperoxidation activity

For these studies, we used hisPON1 complexed with uHDL and then measured its ability to degrade lipid peroxides generated by tBHP. For these studies, 50 μg of uHDL was incubated either alone or with 0.5 μg hisPON1 in total 50-μl reaction mixture (1 mg uHDL/ml) containing 10 mM PBS, pH 7.5, and 1 mM CaCl_2_ for 1 h at 37 °C. After incubation, DMSO or 1.6 mol eq IsoLG was added to samples containing uHDL-hisPON1, and all samples were further incubated for 24 h. On the following day, samples were diluted in PBS and then 1 μl of either buffer or 10 mM, and 50 mM tBHP (final concentrations of 100 μM and 500 μM, respectively) were added, followed by vortexing for 5 s. To quantitatively measure the antiperoxidative activity of HDL, 1 μl of 10 mM TM10-H was added and vortexed, and samples were further incubated for 30 min at 37 °C and then subjected to EPR. TM10-H is a lipophilic derivative of previously characterized cyclic hydroxylamine spin probes (54) and reacts rapidly with alkyl peroxyl radicals to produce a stable nitroxide radical that can be quantified by EPR (Fig. S2).

### Effect of IsoLG adducts on PON1 hyperactivation

Twenty microgram of 1 mg/ml uHDL (400 μg/ml) was preincubated with increasing concentrations of IsoLG (0–5 mol eq to apoA-I) in total 50 μl of lactonase assay buffer for 1 h (for reversible IsoLG adducts) and 24 h (for irreversible IsoLG adducts). Then, 0.2 μg hisPON1 (10 μl) was added for another 1 h. Samples were diluted in 40-μl assay buffer, and the percentage enzymatic activity was measured with DTNB and TBBL as described above.

### Enzyme kinetic measurements

Inhibition kinetics of hisPON1 with IsoLG was determined with TBBL. 0.0075 μM hisPON1 (10 μl) was incubated with different IsoLG concentrations (0–50 μM) in 145 μl of 10 mM PBS (pH 7.5) containing 1 mM calcium chloride and 0.8 mM DTNB. Final concentration of DMSO was 0.5% in all wells. Forty microliter TBBL (final concentration 0–2 mM) was added to the reaction mixture and its hydrolysis monitored at 412 nm for 45 min at 37 °C. TBBL was freshly prepared in 10 mM PBS (pH 7.5) containing 1 mM calcium chloride. The averaged initial slope measurements for hisPON1 activity in the absence of IsoLG was set as 100% activity and the normalized percentage activity calculated by dividing the initial slope observed with each concentration of IsoLG by this value. Fitting of the response curve and calculation of the IC_50_ and 95% confidence interval was performed using log(inhibitor) *versus* normalized response –variable slope analysis in GraphPad Prism version 7.04.

### PON1 pulldown assay

Fifty microgram of uHDL (1 mg/ml final) was incubated with 2.9 μg hisPON1 for 1 h in total 50 μl PBS buffer (10 mM PBS, pH 7.5, 1 mM CaCl_2_). This complex was then treated with or without MPO for 24 h. On the following day, samples were diluted with 950 μl PBS and then treated with 0.1% NP-40, vortexed for 5 s, and further incubated at 25 °C on an orbital shaker at 1500 rpm for 2 h. After treatment, 25 μl of Ni-beads were added to each sample followed by incubation at 40 °C for 30 min. Samples were then centrifuged at high speeds to pellet down beads. Ni-beads were washed twice with 1 ml PBS and then with buffer A (PBS containing 0.5 M NaCl and again with PBS). All supernatants were combined and saved. Finally, beads were reconstituted in 200 μl of PBS. Both beads and supernatant were used for IsoLG–Lys measurements by LC/MS.

### Statistical analysis

In all reactions, the IC_50_ was calculated using log(inhibitor) *versus* normalized response variable slope analysis in GraphPad Prism version 7.04.

## Data availability

The datasets generated during and/or analyzed during the present study are available on reasonable request from the corresponding author, Dr Sean S. Davies at Vanderbilt University, sean.davies@vanderbilt.edu.

## Supporting information

This article contains supporting information.

## Conflict of interest

The authors declare that they have no conflicts of interest with the contents of this article.
