# Supplementary material for: Myeloperoxidase-induced modification of HDL by isolevuglandins inhibits paraoxonase-1 activity
Source: J Biol Chem. 2021 Jul 29;297(3):101019. doi: 10.1016/j.jbc.2021.101019 (PMC8390528; doi:10.1016/j.jbc.2021.101019)
Supplement: Supplemental Figures S1–S5 [file mmc1.pdf]

Supporting Information

Myeloperoxidase-induced modification of HDL by isolevuglandins inhibits paraoxonase activity

Geetika Aggarwal[1], Linda S. May-Zhang[1], Valery Yermalitsky[1], Sergey Dikalov[2], Maxim A. Voynov[5], Venkataraman Amaranth[2], Valentina Kon[3], MacRae F. Linton[1,4], Kasey C. Vickers[4], Sean S. Davies[1,2].

[1]Department of Pharmacology, Vanderbilt University, Nashville, Tennessee, USA. [2]Department of Medicine, Division of Clinical Pharmacology; [3]Department of Pediatrics, Division of Nephrology and Hypertension; [4]Department of Medicine, Division of Cardiovascular Medicine, Atherosclerosis Research Unit, Vanderbilt University Medical Center, Nashville, Tennessee, USA. [5]North Carolina State University, Raleigh, North Carolina, USA.

Supporting Methods

Synthesis of 5-thiobutyl butyrolactone (TBBL). To a mixture of $AlBr_3$ (5 g) and butanethiol (15 ml) γ-butyrolactone was added dropwise. The turbid mixture, on stirring for 2 h, became clear. It was slowly poured into ice-cold water (35 ml) and the aqueous solution was extracted with CH_2Cl_2 (2 × 35 mL). The extracts were combined and dried. CH_2Cl_2 and excess butanethiol were sequentially removed. TLC (4:1 hexane-ethyl acetate): R_F 0.8. 4-Butylthiobutyric acid was cooled in ice and a cold solution (21 ml) of 0.5 M $NaIO_4$ (2.24 g) was added. The mixture was stirred at 4 °C for 18 h. The solid was removed and the filtrate was evaporated at 50-55 °C and ~10 Torr. The residue was stirred with CH_2Cl_2 (40 mL) for 15 m, let settle and the supernatant liquid was removed. Extraction was repeated twice more, and the combined extracts were dried and concentrated to a clear syrup (2 g). The sulfoxide (1 g) was dissolved in toluene (30 ml), mixed with acetic anhydride (2 ml) and *p*-toluenesulfonic acid (a few crystals), and refluxed for 1 h. After cooling, toluene was removed, and the residue was purified by column chromatography (silica; hexane followed by 4:1 and 3:1 hexane-ethyl acetate) to get the title compound; (0.32 g).

Synthesis of 4-Decyloxy-1-hydroxy-2,2,6,6-tetramethylpiperidine hydrochloride (TM10-H). TM10-H was synthesized by reduction of the corresponding nitroxide (4-decyloxy-2,2,6,6-tetramethylpiperidine 1-oxyl) with a mixture of aqueous HCl and ethanol. This process is a combination of an acid-driven disproportionation of a nitroxide and a hydride ion transfer from ethanol to the oxoammonium cation which is an oxidized form of the nitroxide.

Comparison of PON1 activity in dHDL and uHDL. 40 µg each of dHDL and three different uHDL samples were diluted in lactonase assay buffer (10mM PBS pH 7.5, 1mM $CaCl_2$) and 10 µl of 10 mM DTNB (0.8mM final concentration), and then 10 µL of TBBL (1 mM final concentration) added in total 120 µl reaction mixture and hydrolysis by endogenous PON1 was measured as change in absorbance at 405 nm in Synergy H1 plate reader (Supplement Figure 1).

Development of anti-peroxidation activity assay. To identify appropriate concentrations of tert-butylhydroperoxide (tBHP) needed to generate sufficient lipid peroxyl radicals to measure PON1 anti-peroxidation activity, 50 µg of uHDL was incubated either alone or with 0.5µg hisPON1 in total of 50 µl reaction mixture containing PBS buffer (10 mM PBS pH 7.5 and 1 mM $CaCl_2$) for 1 h at 37^0C. After incubation, samples were diluted in PBS buffer and then 1 µl of 5 mM, 50 mM and 100 mM tert-butylhydroperoxide (tBHP) added (final concentration 50 µM, 500 µM and 1000 µM respectively), followed by 1 µl of 100 mM T_{10M-H} probe (1 mM final concentration). Samples were vortexed and incubated for 30 min at 37^0C and then analyzed by EPR. To determine the peroxide content of individual agents, buffer only, buffer containing uHDL and buffer containing uHDL and hisPON1 were prepared in the same way, without adding tBHP. All incubation tubes were wrapped in aluminum foil to prevent light-induced degradation of probe. Working stocks of tBHP were freshly prepared each day in double distilled water.

Based on these results, 100 µM and 500 µM tBHP were chosen as appropriate final concentrations at which to test the effect of IsoLG modification.

Filtrate experiment for detection of free IsoLG: 60 µg of 1mg/ml uHDL (400 µg/ml) alone or uHDL was pre-incubated with 5 mol eq IsoLG for 1 h and 24 h at 37^0C in total 150 µl reaction mixture. IsoLG alone was used as a positive control. After incubation, samples were diluted in 2500 µl 10mM PBS pH 7.5 and filtered with 10 kDa centrifugal filter column by using centrifugation at 3200 g for 20 min. After centrifugation, both left over uHDL (on the top) and filtrate (on the bottom) were collected. Protein concentration was measured in left over uHDL. To the filtrate, 0.3 mM lysine was added for 24 h and different IsoLG-Lys adducts were measured by LC-MS as described previously.

Optimization of PON1 pulldown assay. 50 µg of uHDL (1mg/ml final) incubated with 2.9 µg hisPON1 for 1 h in total 50 µl PBS buffer (10 mM PBS pH 7.5, 1 mM $CaCl_2$) and after incubation, samples were diluted with 950 µl PBS buffer. Samples then treated with either 0%, 0.1% NP-40, 0.5% NP-40, vortexed for 5 sec and were further incubated at 25^0C on orbital shaker at 1500 rpm for 2 h. After treatment, 25 µl of Ni-beads were added to each sample followed by incubation at 4^0C for 30 min. Samples then centrifuged and beads were washed twice with 1 ml PBS buffer. After that half of samples washed with buffer A (PBS buffer containing 0.5 M NaCl) and other half similar set of samples with 1 ml PBS buffer. Finally, beads were reconstituted in 200 µl of PBS buffer and used for PON1 and ApoA1 immunoblot analysis.

Supporting Figures

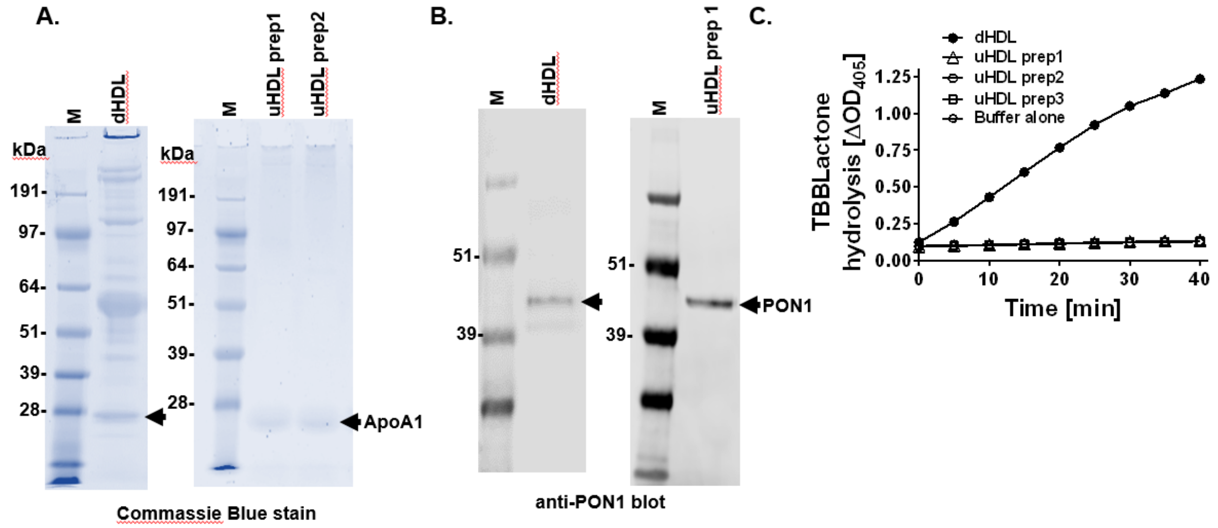

Supplemental Figure 1. Effects of isolation procedures on purity of HDL and PON1 activity. A) Commassie Blue stain of dextran-isolated HDL(dHDL) and density-gradient ultracentrifugation-isolated HDL preparations (uHDL) preparations run on SDS-PAGE. B) anti-PON1 immunoblot of uHDL and dHDL preparations. C) Lactonase activity of dHDL and uHDL preparations.

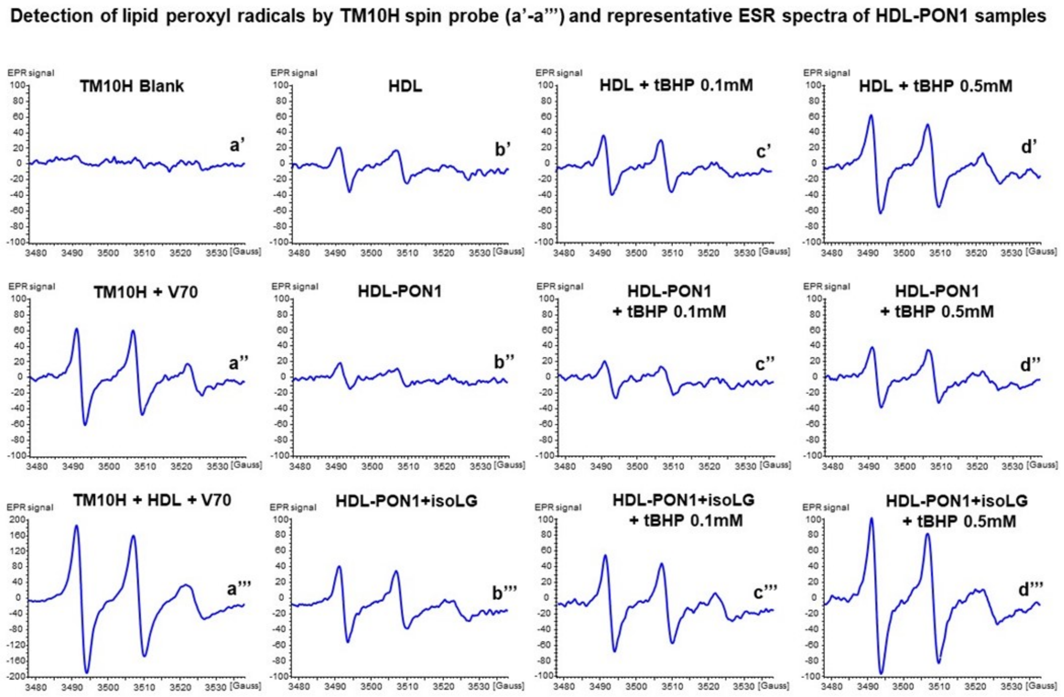

Supplemental Figure 2. Detection of lipid peroxyl radicals by lipophilic TM10H spin probe (a'-a''') and representative EPR spectra of hisPON-uHDL samples (b-d). Cyclic hydroxylamine spin probe TM10H is ESR silent (a') but reacts with alkyl peroxyl radicals generated by radical initiator V70 (a'') or lipid peroxyl radicals produced by V70 plus HDL (a''') yielding TM10 nitroxide radical revealed by appearance of three-line ESR spectra (a''-a'''). To quantitatively measure the anti-peroxidative activity of HDL, TM10H probe (0.1mM) was incubated with HDL samples for 30min at 37°C. Then samples were placed into 50 µl glass micropipettes, and EPR spectra were recorded at room temperature using EMX EPR spectrometer (Bruker Biospin Corp., Billerica, MA). The EPR settings were as follows: field sweep, 60 Gauss; microwave frequency, 9.84 GHz; microwave power, 20 milliwatts; modulation amplitude, 2 Gauss; scan time, 80 sec; time constant, 1.3 sec; and receiver gain, 50 dB. Figures (b-d) show representative EPR spectra of samples containing TM10H spin probe, uHDL, hisPON1, IsoLG-treated uHDL-hisPON1 and tert-butyl hydroperoxide (tBHP).

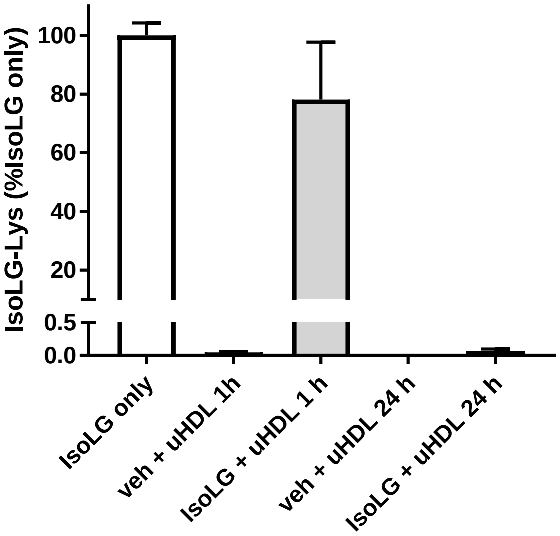

Supplemental Figure 3. Quantitation of IsoLG still available to react with PON1 after reaction of 5 mol eq IsoLG with density gradient ultracentrifugation-isolated HDL (uHDL) for either 1 h or 24 h. As a control, 5 mol eq IsoLG only was incubated in buffer for 1 h (n = 2 replicates per condition). After incubation for stated time, each reaction was filtered with 10 kDa cutoff centrifugal filter. To detect free IsoLG in filtrate, filtrate was incubated with 0.3 mM L-lysine for 24 h and resulting IsoLG-Lys adducts (lactam, hydroxylactam, pyrrole, and their anhydro forms) detected by LC-MS.

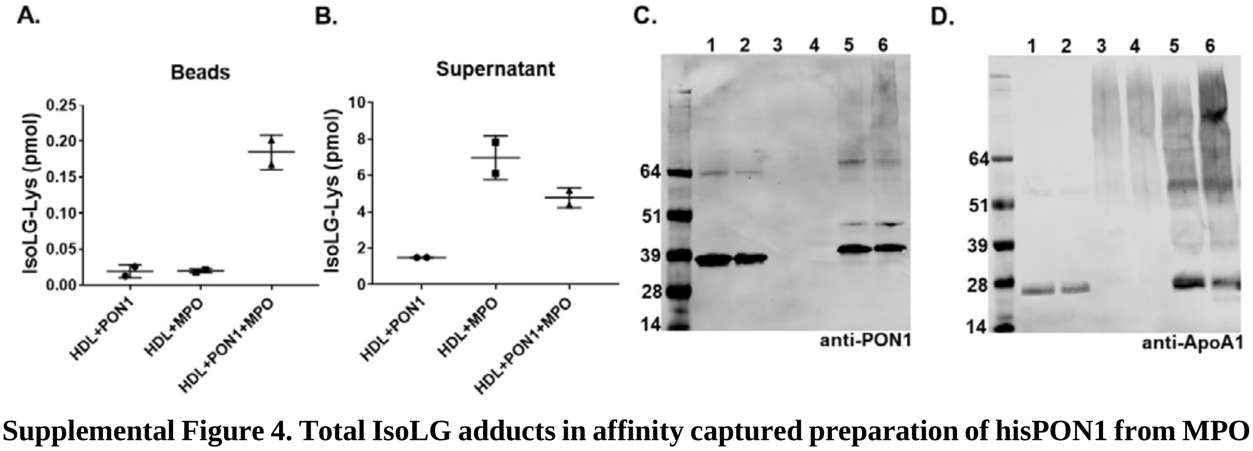

Supplemental Figure 4. Total IsoLG adducts in affinity captured preparation of hisPON1 from MPO treated hisPON1-uHDL. 75 µg uHDL only or with 4.2 µg hisPON1 incubated with MPO oxidase system for 24h. After 24h, hisPON1 pool down using nickel beads. IsoLG-lys levels in beads and supernatant measured by LC-MS. Levels of IsoLG-Lys on hisPON1 recovered by A) nickel bead precipitation and in B) supernatant after nickel bead precipitation from hisPON1-uHDL incubated with and without MPO C) PON1 immunoblot of precipitated hisPON1 from uHDL with nickel beads after stripping with 10mM PBS buffer pH 7.5 containing 0.1%NP-40 could not fully remove co-precipitated proteins such as D) ApoA1. Lanes 1-2: HDL+PON1; Lanes 3-4: HDL+MPO; Lanes 5-6: HDL + PON1+MPO.

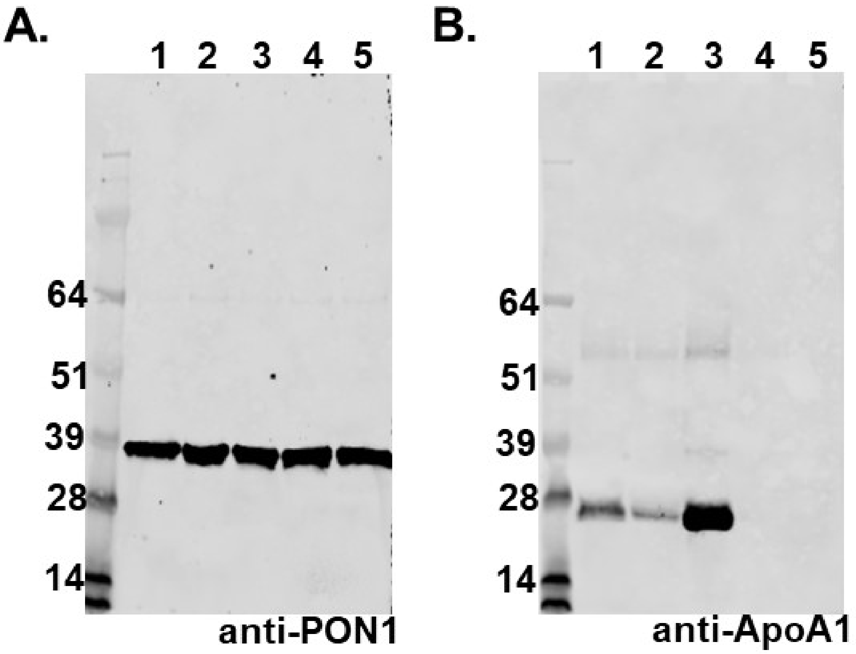

Supplemental Figure 5. Optimization of hisPON1 affinity purification to minimize the quantity of ApoA1 retained with hisPON1. 50 µg uHDL was incubated with 2.9 µg hisPON1 for 1 h, then mixed with 25 µl of Ni-beads to capture hisPON1. Beads were washed with PBS buffer containing 0.1% NP-40 (lane 1), 0.5% NP-40 (lane 2), 0.5M NaCl (lane 3), 0.1% NP-40 and 0.5M NaCl (lane 4) and 0.5% NP-40 and 0.5M NaCl (lane 5) two times followed by only PBS buffer. Beads then reconstituted in 200 µl PBS buffer, mixed with 5X SDS-PAGE sample buffer, heated at 80^0C for 10 min, proteins separated by reducing 4-20% SDS-PAGE gels, and proteins transferred to membranes for immunoblotting with (A) anti-PON1 antibody or (B) anti-apoA-I antibody.
